# Supplementary material for: Abdominal volume index, waist-to-height ratio, and waist circumference are optimal predictors of cardiometabolic abnormalities in a sample of Lebanese adults: A cross-sectional study
Source: PLOS Glob Public Health. 2023 Dec 21;3(12):e0002726. doi: 10.1371/journal.pgph.0002726 (PMC10734963; doi:10.1371/journal.pgph.0002726)
Supplement: S2 Fig — (DOCX) [file pgph.0002726.s002.docx]

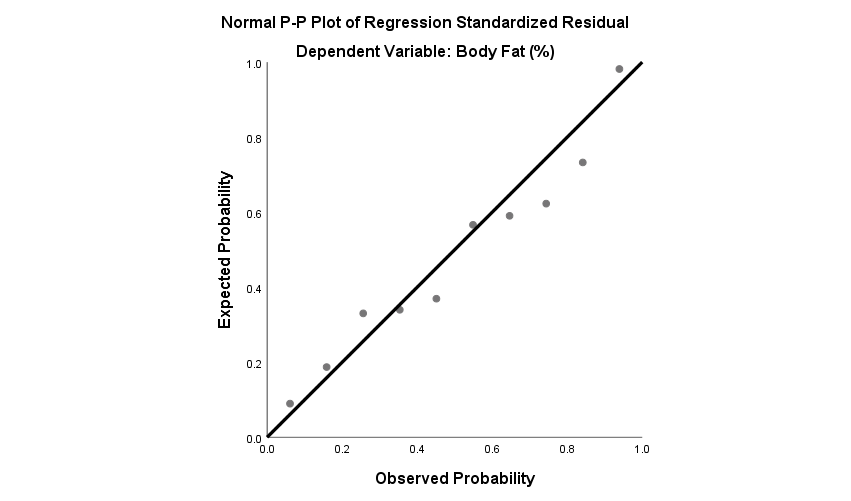

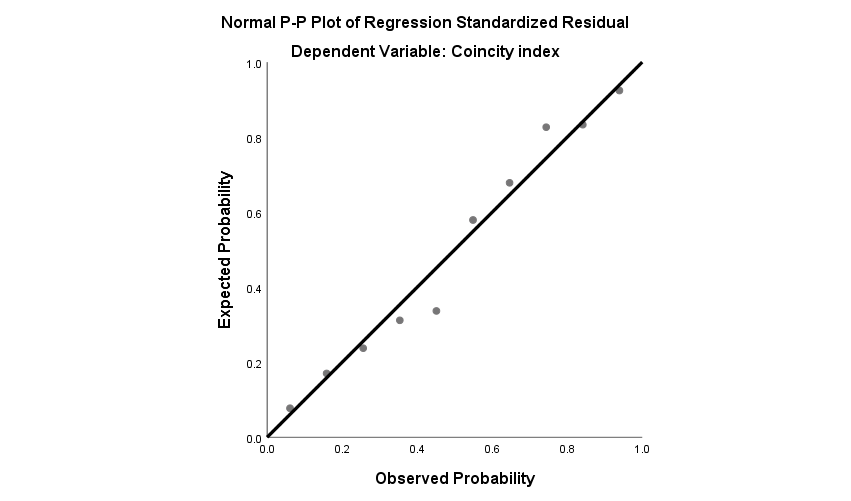

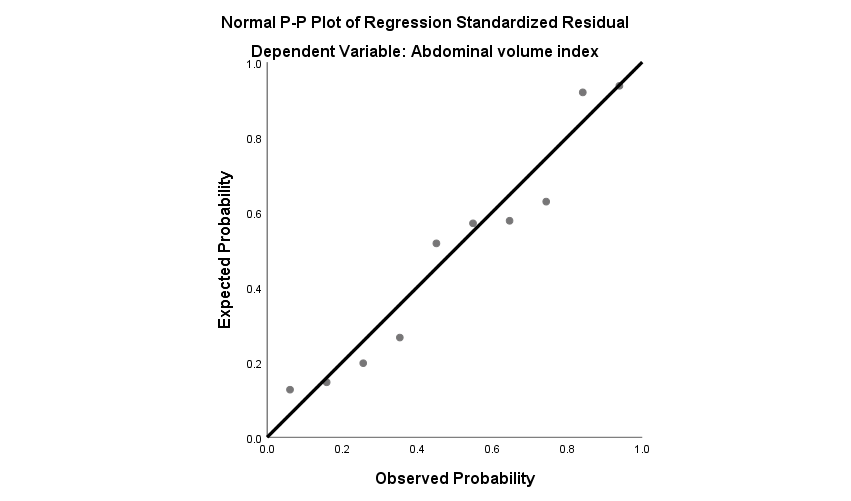

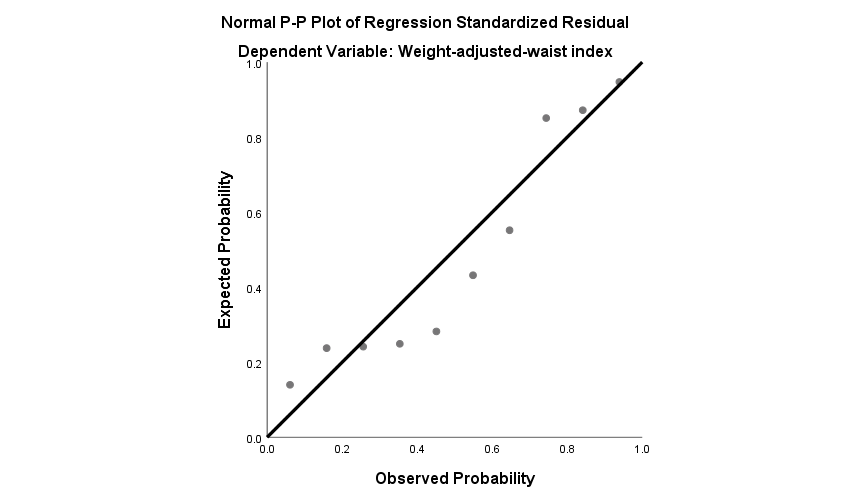


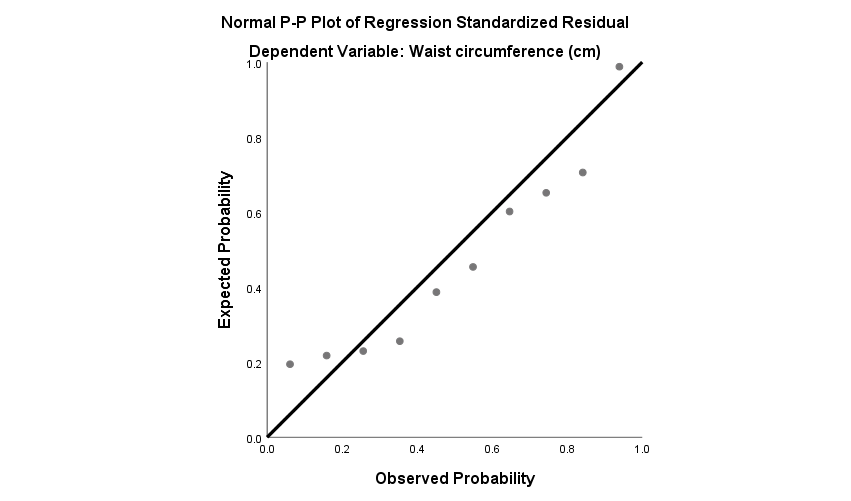

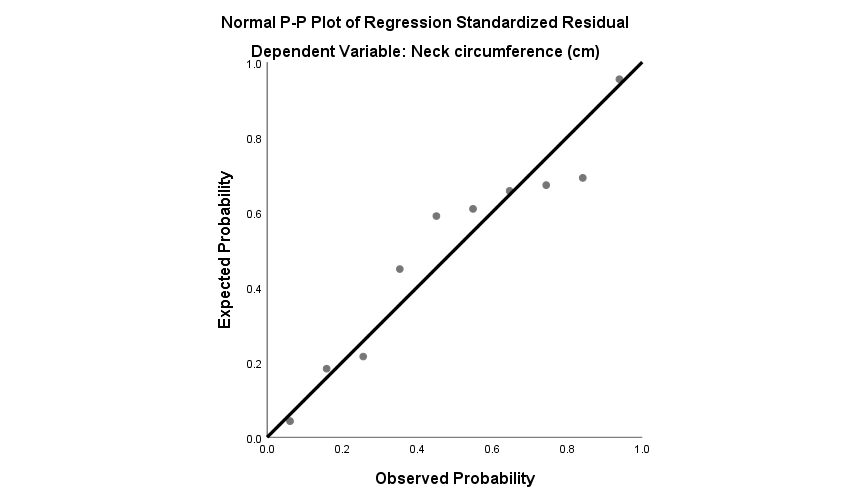

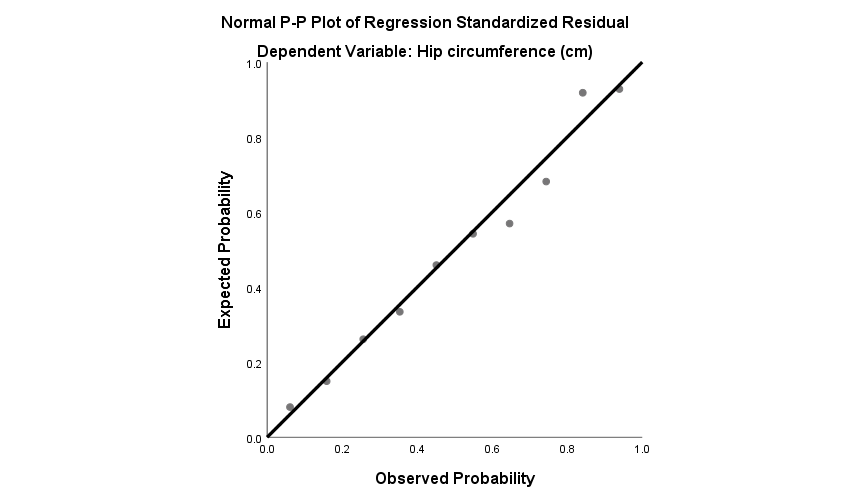

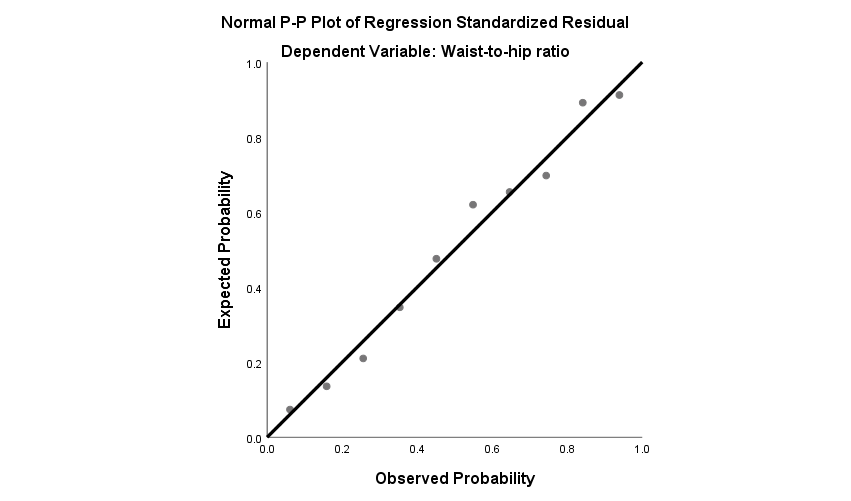


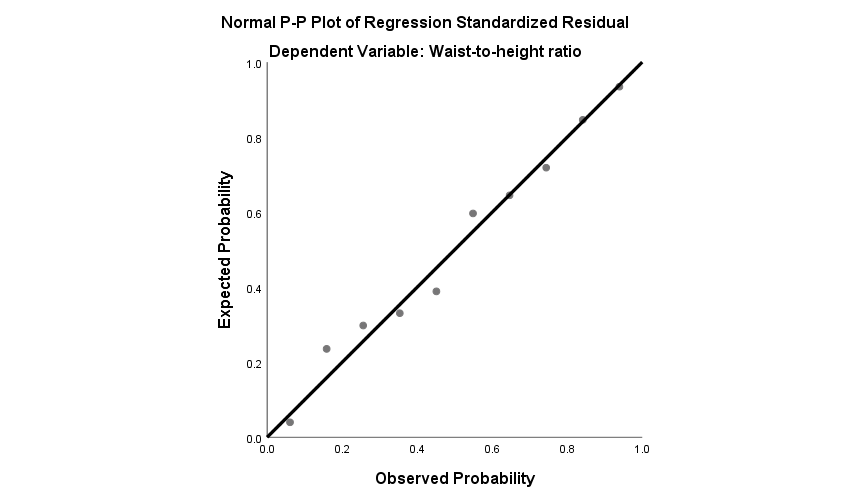

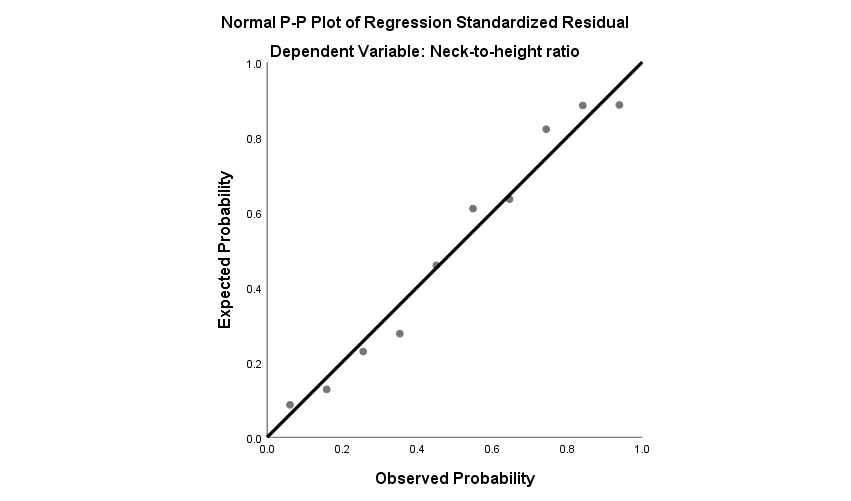

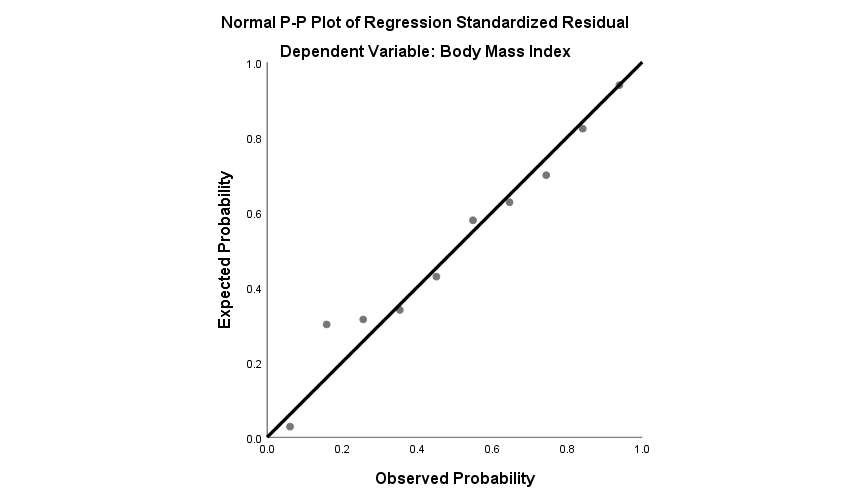


**S2 Fig:** Calibration plot of the anthropometric measures based on the contingency table for Hosmer-Lemeshow statistic among males
