# Supplementary material for: Abdominal volume index, waist-to-height ratio, and waist circumference are optimal predictors of cardiometabolic abnormalities in a sample of Lebanese adults: A cross-sectional study
Source: PLOS Glob Public Health. 2023 Dec 21;3(12):e0002726. doi: 10.1371/journal.pgph.0002726 (PMC10734963; doi:10.1371/journal.pgph.0002726)
Supplement: S1 Table — (DOCX) [file pgph.0002726.s003.docx]

| **S1 Table: The area under the curve of each anthropometric indices for the presence of CMA in the total sample and both genders (N=221).** | | | |
| --- | --- | --- | --- |
|  | **Total sample** | **Females** | **Males** |
| Total body fat percent | 0.662 (0.590; 0.735) | 0.740 (0.656; 0.823) | 0.767 (0.663; 0.871) |
| Conicity index | 0.768 (0.706; 0.831) | 0.763 (0.684; 0.843) | 0.748 (0.632; 0.864) |
| Abdominal volume index | 0.817 (0.760; 0.873) | 0.824 (0.753; 0.895) | 0.788 (0.689; 0.887) |
| Weight-adjusted-waist index | 0.762 (0.698; 0.826) | 0.779 (0.702; 0.856) | 0.737 (0.617; 0.857) |
| Waist circumference (cm) | 0.816 (0.759; 0.873) | 0.823 (0.751; 0.894) | 0.790 (0.691; 0.889) |
| Neck circumference (cm) | 0.676 (0.604; 0.747) | 0.655 (0.563; 0.747) | 0.692 (0.575; 0.810) |
| Hip circumference (cm) | 0.725 (0.657; 0.793) | 0.725 (0.639; 0.812) | 0.738 (0.629; 0.847) |
| Waist-to-hip ratio | 0.771 (0.708; 0.834) | 0.774 (0.695; 0.853) | 0.732 (0.615; 0.849) |
| Waist-to-height ratio | 0.817 (0.761; 0.874) | 0.832 (0.762; 0.902) | 0.789 (0.689; 0.890) |
| Neck-to-height ratio | 0.688 (0.617; 0.759) | 0.694 (0.605; 0.782) | 0.685 (0.567; 0.803) |
| Body Mass Index | 0.770 (0.707; 0.834) | 0.780 (0.701; 0.860) | 0.764 (0.658; 0.870) |
