# Supplementary material for: Abdominal volume index, waist-to-height ratio, and waist circumference are optimal predictors of cardiometabolic abnormalities in a sample of Lebanese adults: A cross-sectional study
Source: PLOS Glob Public Health. 2023 Dec 21;3(12):e0002726. doi: 10.1371/journal.pgph.0002726 (PMC10734963; doi:10.1371/journal.pgph.0002726)
Supplement: S2 Table — (DOCX) [file pgph.0002726.s004.docx]

| **S2 Table: calibration in the presence of CMA in the overall sample and stratified by sex** | | | |
| --- | --- | --- | --- |
|  | **Expected cases ^a^**  **n** | **Observed cases**  **n** | **E/O ratio** |
| **Total sample** |  |  |  |
| Total body fat percent | 121 | 111 | 1.09 |
| Conicity index | 117 | 111 | 1.05 |
| Abdominal volume index | 113 | 107 | 1.05 |
| Weight-adjusted-waist index | 117 | 111 | 1.05 |
| Waist circumference (cm) | 116 | 111 | 1.04 |
| Neck circumference (cm) | 115 | 111 | 1.03 |
| Hip circumference (cm) | 114 | 107 | 1.06 |
| Waist-to-hip ratio | 116 | 107 | 1.08 |
| Waist-to-height ratio | 117 | 111 | 1.05 |
| Neck-to-height ratio | 113 | 111 | 1.01 |
| BMI | 118 | 111 | 1.06 |
| **Females** |  |  |  |
| Total body fat percent | 66 | 64 | 1.03 |
| Conicity index | 66 | 64 | 1.03 |
| Abdominal volume index | 66 | 62 | 1.06 |
| Weight-adjusted-waist index | 66 | 64 | 1.03 |
| Waist circumference (cm) | 65 | 64 | 1.01 |
| Neck circumference (cm) | 65 | 64 | 1.01 |
| Hip circumference (cm) | 64 | 62 | 1.03 |
| Waist-to-hip ratio | 67 | 62 | 1.08 |
| Waist-to-height ratio | 66 | 64 | 1.03 |
| Neck-to-height ratio | 67 | 64 | 1.04 |
| BMI | 67 | 64 | 1.04 |
| **Males** |  |  |  |
| Total body fat percent | 50 | 47 | 1.06 |
| Conicity index | 54 | 47 | 1.14 |
| Abdominal volume index | 49 | 45 | 1.08 |
| Weight-adjusted-waist index | 54 | 47 | 1.14 |
| Waist circumference (cm) | 50 | 47 | 1.06 |
| Neck circumference (cm) | 55 | 47 | 1.17 |
| Hip circumference (cm) | 48 | 45 | 1.06 |
| Waist-to-hip ratio | 46 | 45 | 1.02 |
| Waist-to-height ratio | 49 | 47 | 1.04 |
| Neck-to-height ratio | 50 | 47 | 1.06 |
| BMI | 47 | 47 | 1.00 |
| E/O, ratio of expected to observed cases  ^a^ Expected and observed cases were derived from the classification table in the logistic regression model | | | |
