# Supplementary material for: Abdominal volume index, waist-to-height ratio, and waist circumference are optimal predictors of cardiometabolic abnormalities in a sample of Lebanese adults: A cross-sectional study
Source: PLOS Glob Public Health. 2023 Dec 21;3(12):e0002726. doi: 10.1371/journal.pgph.0002726 (PMC10734963; doi:10.1371/journal.pgph.0002726)
Supplement: Attachment — Submitted filename: PGPH-D-23-00803.pdf [file pgph.0002726.s006.pdf]

PLOS Global Public Health

Abdominal volume index, waist-to-height ratio, and waist circumference are optimal predictors of cardiometabolic abnormalities in a sample of Lebanese adults: A cross-sectional study

--Manuscript Draft--

Manuscript Number:	PGPH-D-23-00803
Article Type:	Research Article
Full Title:	Abdominal volume index, waist-to-height ratio, and waist circumference are optimal predictors of cardiometabolic abnormalities in a sample of Lebanese adults: A cross-sectional study
Short Title:	Waist-to-height and waist circumference best predict cardiometabolic abnormalities
Corresponding Author:	Myriam Abboud<br>Zayed University<br>Dubai, Dubai UNITED ARAB EMIRATES
Order of Authors:	Myriam Abboud
	Suzan Haidar
	Nadine Mahboub
	Dimitrios Papandreou
	Rana Rizk
Keywords:	Anthropometry; Syndrome, Cardiometabolic; ROC Curve

Abstract:

Background

The prevalence of cardiometabolic abnormalities is high globally. This is a cause of concern since these abnormalities increase the risk of morbidity and mortality. Consequently, appropriate screening of utmost importance. Noninvasive, low-cost, and ethnic-specific anthropometric tools are crucial for widespread screening and early detection of cardiometabolic abnormalities.

Methods

221 Lebanese participants enrolled in this cross-sectional study (62.9% females; mean age: 43.36 ± 16.05 years; mean body mass index (BMI): 28.43 ± 6.10 Kg/m2). The main outcome measure was cardiometabolic abnormality (CMA), defined as the presence of at least two or more non-anthropometric components of the Metabolic Syndrome. Several anthropometric indices: Total body fat percent, Conicity index, Abdominal volume index (AVI), Weight-adjusted-waist index, Waist circumference (WC), Neck circumference, Hip circumference, Waist-to-hip ratio, Waist-to-height ratio (WtHR), Neck-to-height ratio, and BMI were assessed in their prediction of CMA, using the area under the receiver-operating characteristic curve (AUC) and 95% confidence intervals (CIs), as well sensitivity, specificity, and negative and positive predictive values measures.

Results

The prevalence of CMA was 52.0% (47.5% in females and 59.8% in males). Significant associations were found between all the anthropometric measures and CMA. AVI, WHtR, and WC were almost equally predictive for CMA in the total sample (AUC: 0.81) and in both genders, with suggested cut-off values of 19.61 and 16.75, 0.58 and 0.56, and 91.25 cm and 101.5 cm among males and females, respectively. Total body fat percent, neck circumference, and neck-to-height had the lowest AUCs for CMA in the total sample and in both genders.

Conclusions

	AVI, WHtR, and WC best predicted CMA in a sample of Lebanese adults. These less invasive, low-cost, easy-to-measure indices can be used to screen widely for CMA to better manage and prevent disease and subsequent morbidity and mortality.
Additional Information:	
**Question**	**Response**
**Financial Disclosure**<br><br>Enter a financial disclosure statement that describes the sources of funding for the work included in this submission and the role the funder(s) played. This includes grants and any commercial funding of the work or authors.<br><br>This statement will be typeset if the manuscript is accepted for publication.<br><br>*Please review the submission guidelines and the instructions link below for detailed requirements and guidance.*	this article was funded by the the grant RIF R21062 from Zayed University
**Competing Interests**<br><br>On behalf of all authors, disclose any competing interests that could be perceived to bias this work.<br><br>This statement will be typeset if the manuscript is accepted for publication.<br><br>*Please review the instructions link below and PLOS Global Public Health's competing interests policy to determine what information must be disclosed at submission.*	No Competing interest
**Data Availability**<br><br>Before publication, Authors are required to make fully available and without restriction all data underlying their findings. Please see our PLOS Data Policy page for detailed information on this policy.	All data are available and kept safely and confidentially with corresponding author

Powered by Editorial Manager® and ProduXion Manager® from Aries Systems Corporation

A **Data Availability Statement**, detailing where the data can be accessed, is required at first submission. Insert your Data Availability Statement in the box below.<br><br>Please see the data reporting section of our submission guidelines for instructions on what you need to include in your Data Availability Statement.<br><br>This statement will be typeset if the manuscript is accepted for publication.<br><br>*PLOS allows rare exemptions to address legal and ethical concerns. If you have legal or ethical restrictions, please use the box below to detail these in full sentences for the Journal team to consider.*	

Manuscript

Click here to access/download;Manuscript;Anthrop and MS-2023-04-29.docx

Abdominal volume index, waist-to-height ratio, and waist circumference are optimal predictors of cardiometabolic abnormalities in a sample of Lebanese adults: A cross-sectional study

Myriam Abboud [1]*, Suzan Haidar [2], Nadine Mahboub [2], Dimitrios Papandreou [3], and Rana Rizk [4,5]

[1] College of Natural and Health Sciences, Zayed University, Dubai, United Arab Emirates; myriam.abboud@zu.ac.ae

[2] Department of Nutrition and Food Sciences, Lebanese International University, Beirut, Lebanon; suzan.haidar@liu.edu.lb, ORCID: 0000-0001-7117-599X; nadine.baltagi@liu.edu.lb, ORCID: 0000-0001-5322-7661

[3] College of Natural and Health Sciences, Zayed University, Abu Dhabi, United Arab Emirates; papandreoudimitrios@yahoo.gr, ORCID: 0000-0002-4923-484X

[4] Department of Natural Sciences, Lebanese American University, Byblos, Lebanon; rana.rizk01@lau.edu.lb; ORCID: 0000-0002-8850-6502

[5] Institut National de Santé Publique, d'Epidémiologie Clinique, et de Toxicologie (INSPECT-LB)

***** Correspondence: myriam.abboud@zu.ac.ae

Short title: Waist-to-height and waist circumference best predict cardiometabolic abnormalities

Abstract

Background

The prevalence of cardiometabolic abnormalities is high globally. This is a cause of concern since these abnormalities increase the risk of morbidity and mortality. Consequently, appropriate screening of utmost importance. Noninvasive, low-cost, and ethnic-specific anthropometric tools are crucial for widespread screening and early detection of cardiometabolic abnormalities.

Methods

221 Lebanese participants enrolled in this cross-sectional study (62.9% females; mean age: 43.36 ± 16.05 years; mean body mass index (BMI): 28.43 ± 6.10 Kg/m^2). The main outcome measure was cardiometabolic abnormality (CMA), defined as the presence of at least two or more non-anthropometric components of the Metabolic Syndrome. Several anthropometric indices: Total body fat percent, Conicity index, Abdominal volume index (AVI), Weight-adjusted-waist index, Waist circumference (WC), Neck circumference, Hip circumference, Waist-to-hip ratio, Waist-to-height ratio (WtHR), Neck-to-height ratio, and BMI were assessed in their prediction of CMA, using the area under the receiver-operating characteristic curve (AUC) and 95% confidence intervals (CIs), as well sensitivity, specificity, and negative and positive predictive values measures.

Results

The prevalence of CMA was 52.0% (47.5% in females and 59.8% in males). Significant associations were found between all the anthropometric measures and CMA. AVI, WHtR, and WC were almost equally predictive for CMA in the total sample (AUC: 0.81) and in both genders, with suggested cut-off values of 19.61 and 16.75, 0.58 and 0.56, and 91.25 cm and 101.5 cm among

males and females, respectively. Total body fat percent, neck circumference, and neck-to-height had the lowest AUCs for CMA in the total sample and in both genders.

Conclusions

AVI, WHtR, and WC best predicted CMA in a sample of Lebanese adults. These less invasive, low-cost, easy-to-measure indices can be used to screen widely for CMA to better manage and prevent disease and subsequent morbidity and mortality.

Keywords

Anthropometry; Syndrome, Cardiometabolic; ROC Curve; Lebanon.

Introduction

Cardiometabolic abnormalities, i.e., hyperglycemia, elevated blood pressure, high triglyceride levels, low levels of low-density lipoprotein (HDL-C), and obesity (1) have long been associated with a higher risk of morbidity and mortality (2, 3).

Globally, the incidence of clustered cardiometabolic abnormalities, such as the metabolic syndrome, is rising due to alterations in the lifestyle, dietary choices, and stress exposure (4-6), with global estimates ranging from 10% to 84% depending on the diagnostic criteria used, age, gender, and ethnicity (7-9). Early diagnosis of clustered cardiometabolic abnormalities is crucial for the adoption of lifestyle changes and lowering disease-related risk factors and optimizing treatment (10, 11), necessitating invasive and costly laboratory measurements of the plasma lipid and glycemic state (12).

Long standing evidence pinpoints excessive visceral fat as the main culprit behind the development of cardiometabolic abnormalities (13). However, methods for measuring it, such as computed tomography and magnetic resonance imaging are expensive and unavailable in routine clinical practice(14). The use of straightforward anthropometric indices of body composition, such as body mass index (BMI), waist circumference (WC), and waist-to-height ratio (WHtR), has been traditionally used as a practical and non-invasive proxy for abdominal adiposity. However, studies found that the association of these indicators with clustered cardiometabolic abnormalities varies amongst populations (15). More sensitive anthropometric indicators that account for body shape may be better predictors of central obesity (13) as individuals with the same WC and BMI might share different risk factors based on differences in statures (16, 17). Several anthropometric indices have been proposed as markers of body fat distribution and predictors of clustered cardiometabolic abnormalities, but without a thorough consensus

concerning the optimal marker and its cut-off values (18-21). The purpose of this study is to examine the relationship between several anthropometric indices, both traditional and novel, and a cluster of cardiometabolic abnormalities in a sample of Lebanese adults, and to identify the optimal index and its cut-off values that best predict cardiometabolic abnormalities.

Materials and Methods

Lebanese adults were invited to participate in this cross-sectional study via community and university announcements. Interested participants were asked to be fasting for at least eight hours on the day of data collection. Inclusion criteria consisted of being aged between 18 and 65 years, of a Lebanese nationality, not diagnosed with a current infection, neither pregnant or lactating, and not being on medications nor suffering from diseases that interfere with vitamin D metabolism. Data were collected between March and June 2022.

Ethical considerations

The study was consistent with the declaration of Helsinki and approved by the Institutional Review Board of the Lebanese International University (Ethical approval no: LIUIRB-220201-SH-111).

All interested participants were approached by trained, qualified research assistants who explained them in detail the objectives of the study and the protocol. All participant were informed in detail about the study and signed a consent form.

Data collection

Sociodemographic characteristics and medical history

A questionnaire was developed to collect data about demographics and medical history. It included questions about age, gender, education, employment, smoking history, and family history of diseases.

Blood withdrawal

A 5 ml of blood was drawn by a licensed phlebotomist from participants in a fasting state after they had been resting for at least 5 minutes in a seated position. Blood was collected into a sterile serum separator tube with a clot activator and then transported to the laboratory for investigation via thermally insulated boxes after which the samples were centrifuged at 4000 revolutions per minute for 10 min and analyzed for blood lipids [total cholesterol (mg/dl), High-Density Lipoprotein-Cholesterol (HDL-C, mg/dl), triglycerides (mg/dl), fasting blood glucose (FBG)(mg/dl)], using an automated chemiluminescence micro-particleimmunoassay (CMIA) kit (ARCHITECT; Abbott Laboratories, Abbott Park, IL, USA). Low-density Lipoprotein-Cholesterol (LDL) was calculated using the Friedwald formula when triglyceride levels were below 400 mg/dl, otherwise they were measured.

Blood Pressure (BP)

After at least 5 minutes of rest and before blood withdrawal, a qualified technician used a standardized mercury sphygmomanometer to take two consecutive reading to measure systolic and diastolic blood pressure (SBP, DBP). Both readings were taken on the same arm where the average of both readings was used for analysis.

Anthropometry

Anthropometric data was collected using standardized techniques and calibrated equipment by trained examiners. Participants were asked to take off their shoes and wear only light clothing during assessment. A portable stadiometer was used to measure height (ADE, Germany) and a

beam scale was used to weigh the participants (kg). Height and weight were taken to the nearest 0.1 cm 100 g respectively while in participants were standing in the Frankfort plane. Body mass index (BMI) was calculated by dividing weight by squared height in meters (m^2). WC (cm) was measured to the nearest 0.1 cm at the mid-point, half-way between the right iliac crest and the lower costal region using standardized measuring tape. Waist-to-height ratio (WHtR) was computed by dividing WC by height (22). Neck circumference (NC-cm) was measured just below the laryngeal prominence perpendicular to the long axis of the neck while the head was positioned in Frankfurt horizontal plane (23). Neck-to-heigh ratio was calculated by dividing neck circumference by height. Hip circumference was measured using measuring tape at the level of the anterior superior iliac spine, at the widest circumference below the waist. Waist-to-hip ratio was then calculated by dividing the WC to hip circumference. Total body fat percent was measured using a multifrequency bioelectrical impedance analysis instrument (InBody 230, InBody, Seoul, Korea). For the bioelectrical impedance analysis, participants were fasting, with empty bladder, standing with both arms 45° apart from the body trunk, and having both feet bared on the spots of the equipment. Total body fat percent was shown on the equipment screen. Conicity index (C index), abdominal volume index (AVI), and weight-adjusted-waist index (WWI) were calculated as follows (12):

$$C - Index = \frac{WC\ (m)}{0.109\sqrt{\frac{body\ weight\ (kg)}{height\ (m)}}}$$

$$AVI = \frac{2\ \times (WC\ (cm))^2 + 0.7\ \times\ (WC\ (cm) - HC(cm))^2}{1000}$$

$$WWI = \frac{WC\ (cm)}{\sqrt{weight\ (kg)}}$$

Definition of Clinical Metabolic Abnormality

A CMA was defined as the presence of two or more of the following non-anthropometric cardiometabolic abnormalities: elevated triglycerides (≥150 mg/dL) or undergoing treatment for it; reduced HDL-C (<40 mg/dL in males and <50 mg/dL in females) or specific treatment for the abnormality; raised BP (SBP ≥130 or DBP≥ 85 mmHg) or undergoing treatment for hypertension; and FBG ≥100 mg/dL or previously diagnosed with diabetes.

Statistical analysis

The data were analyzed using the SPSS software, version 25. A descriptive analysis was done using the counts and percentages for categorical variables and mean and standard deviation for continuous measures. In order to compare between the variables and clinical metabolic abnormality (CMA), the Chi-square and Fisher exact tests were used to compare between categorical variables and the Student t-test was used to compare continuous variables in two groups. We used the area under the receiver-operating characteristic curve (AUC) and 95% confidence intervals (CIs) to assess the discriminatory power of each anthropometric indices to assess the risk for CMA. Also, the sensitivity and specificity as well as the negative and positive predictive values were calculated to assess the probability of the measures to indicate the CMA. Since the anthropometric indices are correlated with sex, the analysis was stratified into two groups (males and females). P-values < 0.05 were considered statistically significant.

Results

Sample description

A total of 221 participants agreed to enroll in this study. Most of the participants were females (62.9%) and more than half of them were married (55.7%) with low socioeconomic income

(50.5%). Almost half of them have a university education level (46.2%) and employed (47.0%). Only 21.3% were cigarette smokers and 31.6% smoke waterpipe. Regarding diseases distribution, 18.1% had diabetes, 28.5% had dyslipidemia, and 57.3% had hypertension. More than half of the participants have a family history of diabetes (54.5%) and hypertension (57.3%), and 38.6% had a family history of dyslipidemia. The mean age of the participants was 43.36 ± 16.05 years. Table 1 shows the sociodemographic and other characteristics of the participants.

Table 1: Sociodemographic and other characteristics of the participants (N=221).

**Variable**	
	**Mean ± SD**
**Age**	43.36 ± 16.05
**Gender**	**N (%)**
Male	82 (37.1%)
Female	139 (62.9%)
**Marital status**	
Single/widowed/divorced	98 (44.3%)
Married	123 (55.7%)
**Education level**	
University degree	102 (46.2%)
High school	41 (18.6%)
Middle education	37 (16.7%)
Primary education	30 (13.6%)
Illiterate	11 (5.0%)
**Socioeconomic status**	

Low	111 (50.5%)
Medium	102 (46.4%)
High	7 (3.2%)
**Employed**	
Yes	103 (47.0%)
No	116 (53.0%)
**Cigarette smoking**	
Never	158 (71.5%)
Previous smoker	16 (7.2%)
Smoker	47 (21.3%)
**Waterpipe smoking**	
Never	129 (58.4%)
Previous smoker	22 (10.0%)
Smoker	70 (31.6%)
**Having Diabetes Mellitus**	
No	172 (77.8%)
Yes, Type 1	3 (1.4%)
Yes, Type 2	33 (14.9%)
Yes, does not know the type	4 (1.8%)
Does not know	9 (4.1%)
**Family history of diabetes**	
No	100 (45.5%)
Yes	120 (54.5%)
**Having disorders of lipid metabolism**	
Yes	63 (28.5%)

No	158 (71.5%)
**Family history of dyslipidemia**	
Yes	85 (38.6%)
No	135 (61.4%)
**Having hypertension**	
Yes	46 (20.8%)
No	175 (79.2%)
**Family history of hypertension**	
Yes	126 (57.3%)
No	94 (42.7%)

Description of anthropometric indices

Table 2 presents the descriptive statistics of the anthropometric indices used in this study.

Table 2: Description of the anthropometric indices in the total sample (N=221).

	**Mean ± SD**	**Median**	**Minimum**	**Maximum**
Total body fat percent	35.54 ± 10.28	36.30	9.40	54.50
Conicity index	1.29 ± 0.13	1.31	0.52	1.67
Abdominal volume index	19.42 ± 6.15	19.30	8.01	37.64
Weight-adjusted-waist index	0.11 ± 0.01	0.11	0.04	0.15
Waist circumference (cm)	96.31 ± 16.58	98.00	44.00	137.00
Neck circumference (cm)	40.40 ± 4.35	40.00	30.00	52.00
Hip circumference (cm)	107.93 ± 12.73	107.80	46.00	146.00
Waist-to-hip ratio	0.89 ± 0.12	0.90	0.36	1.89
Waist-to-height ratio	0.58±0.10	0.59	0.27	0.93

Neck-to-height ratio	0.24±0.02	0.24	0.19	0.32
Body Mass Index	28.43 ± 6.10	27.99	17.42	47.65

More than half of the sample (52.0%) presented CMA; this was more common in males than females (47.5% vs. 59.8%). The association between the anthropometric indices and CMA is presented in Table 3. The results showed that a significant association between all the measures and CMA, in the total sample and when stratifying between gender, whereby significantly higher mean anthropometric indices were found in those having CMA as compared with those who do not have CMA.

Table 3: Association between anthropometric indices and clinical metabolic abnormality (CMA) in the total sample and both genders (N=221).

	**Total sample**		**Among Females**		**Among Males**	
	**Presence of CMA (n=115 (52.0%))**	**Absence of CMA (n=106 (48.0%))**	**Presence of CMA (n=66 (47.5%))**	**Absence of CMA (n=73 (52.50%))**	**Presence of CMA (n=49 (59.80%))**	**Absence of CMA (n=33 (40.20%))**
Total body fat percent	38.10±9.51	32.77±10.42	43.88 ± 6.20	37.27 ± 8.10	30.32±7.41	22.80±7.74
p-value	**<0.001**		**<0.001**		**<0.001**	
Conicity index	1.35±0.09	1.23±0.14	1.34±0.11	1.21±0.15	1.36±0.06	1.28±0.10
p-value	**<0.001**		**<0.001**		**<0.001**	

Abdominal volume index	22.60±5.36	15.97±4.99	22.31±5.77	15.31±4.99	22.99±4.77	17.33±4.76
p-value	**<0.001**		**<0.001**		**<0.001**	
Weight-adjusted-waist index	0.11±0.01	0.10±0.01	0.11±0.01	0.10±0.01	0.11±0.005	0.10±0.009
p-value	**<0.001**		**<0.001**		**<0.001**	
Waist circumference (cm)	104.85±12.99	87.05±15.04	103.95±14.20	84.86±15.38	106.08±11.20	91.88±13.26
p-value	**<0.001**		**<0.001**		**<0.001**	
Neck circumference (cm)	41.64±4.07	39.06±4.27	40.34±3.85	38.27±4.05	43.39±3.71	40.81±4.28
p-value	**<0.001**		**0.002**		**0.005**	
Hip circumference (cm)	111.98±12.43	103.52±11.57	113.21±14.32	104.62±11.74	110.32±9.16	101.22±11.02
p-value	**<0.001**		**<0.001**		**<0.001**	
Waist-to-hip ratio	0.94±0.12	0.84±0.10	0.93±0.15	0.81±0.10	0.96±0.05	0.90±0.09
p-value	**<0.001**		**<0.001**		**0.004**	
Waist-to-height ratio	0.64±0.08	0.53±0.09	0.66±0.09	0.53±0.10	0.61±0.06	0.53±0.08
p-value	**<0.001**		**<0.001**		**<0.001**	

Neck-to-height ratio	0.25±0.02	0.23±0.02	0.25±0.02	0.23±0.02	0.25±0.02	0.23±0.02
p-value	**<0.001**		**<0.001**		**0.004**	
Body Mass Index	30.96±5.76	25.68±5.21	32.04±6.08	25.99±5.64	29.51±5.02	24.98±4.08
p-value	**<0.001**		**<0.001**		**<0.001**	

Table 4 shows the AUCs (and 95%CIs) of anthropometric indices in the prediction of CMA. Overall, AVI, WHtR, and WC were highly and almost equally predictive for CMA in the total sample (AUC: 0.81) (Figure 1) and in both genders (Figures 2 and 3). Total body fat percent, neck circumference, and neck-to-height had the lowest AUCs for CMA in the total sample and in both genders.

Table 4: The area under the curve of each anthropometric indices for the presence of CMA in the total sample and both genders (N=221).

	Total sample	Females	Males
Total body fat percent	0.662 (0.590; 0.735)	0.740 (0.656; 0.823)	0.767 (0.663; 0.871)
Conicity index	0.768 (0.706; 0.831)	0.763 (0.684; 0.843)	0.748 (0.632; 0.864)
Abdominal volume index	0.817 (0.760; 0.873)	0.824 (0.753; 0.895)	0.788 (0.689; 0.887)
Weight-adjusted-waist index	0.762 (0.698; 0.826)	0.779 (0.702; 0.856)	0.737 (0.617; 0.857)

Waist circumference (cm)	0.816 (0.759; 0.873)	0.823 (0.751; 0.894)	0.790 (0.691; 0.889)
Neck circumference (cm)	0.676 (0.604; 0.747)	0.655 (0.563; 0.747)	0.692 (0.575; 0.810)
Hip circumference (cm)	0.725 (0.657; 0.793)	0.725 (0.639; 0.812)	0.738 (0.629; 0.847)
Waist-to-hip ratio	0.771 (0.708; 0.834)	0.774 (0.695; 0.853)	0.732 (0.615; 0.849)
Waist-to-height ratio	0.817 (0.761; 0.874)	0.832 (0.762; 0.902)	0.789 (0.689; 0.890)
Neck-to-height ratio	0.688 (0.617; 0.759)	0.694 (0.605; 0.782)	0.685 (0.567; 0.803)
Body Mass Index	0.770 (0.707; 0.834)	0.780 (0.701; 0.860)	0.764 (0.658; 0.870)

Figure 1. ROC-curves illustrating the discriminatory power of the different anthropometric indices to predict CMA in the total sample.

Figure 2. ROC-curves illustrating the discriminatory power of the different anthropometric indices to predict CMA among females.

Figure 3. ROC-curves illustrating the discriminatory power of the different anthropometric indices to predict CMA among males.

Table 5 shows the measures of diagnostic accuracy of each of the anthropometric indices for the presence of CMA in the total sample and in both genders. In the total sample, the results showed optimal cut-off values of 18.88 for AVI, 0.58 for WHtR, and 94.5 cm for WC. Among females,

optimal cut-off values were 16.75 for AVI, 0.58 for WHtR, and 91.25 cm for WC, and among males, optimal cut-off values were 19.61 for AVI, 0.56 for WHtR, and 101.50 cm for WC.

Table 5: Measures of Diagnostic Accuracy of each anthropometric indices for the presence of CMA in the total sample and both genders (N=221).

In the total sample

	Value	Sensitivity	Specificity	PPV	NPV
Total body fat percent	34.35	70.30	55.90	62.76	62.86
Conicity index	1.28	80.20	61.80	68.97	73.79
Abdominal volume index	18.88	74.80	71.60	73.44	71.60
Weight-adjusted-waist index	0.10	80.20	62.70	69.52	74.10
Waist circumference (cm)	94.50	77.50	70.60	74.06	74.34
Neck circumference (cm)	39.60	73.00	52.90	62.67	64.39
Hip circumference (cm)	106.75	70.30	60.80	66.02	65.39
Waist-to-hip ratio	0.87	79.30	60.80	68.67	73.05
Waist-to-height ratio	0.58	77.50	70.60	74.06	74.34
Neck-to-height ratio	0.23	70.30	53.90	62.29	62.62
Body Mass Index	27.86	72.10	70.60	72.65	70.02

**Among females**					
Total body fat percent	40.20	73.40	62.30	63.01	72.14
Conicity index	1.27	73.40	63.80	63.63	72.46
Abdominal volume index	16.75	81.30	72.50	71.95	81.04
Weight-adjusted-waist index	0.11	70.30	68.10	65.98	71.88
Waist circumference (cm)	91.25	81.30	73.90	73.81	81.37
Neck circumference (cm)	38.25	67.20	52.20	55.99	63.75
Hip circumference (cm)	106.50	73.40	60.90	62.94	71.68
Waist-to-hip ratio	0.86	70.30	66.70	65.64	71.28
Waist-to-height ratio	0.58	81.30	72.50	72.79	81.08
Neck-to-height ratio	0.24	70.30	56.60	59.44	67.81
Body Mass Index	28.14	81.30	72.50	72.79	81.08
**Among males**					
Total body fat percent	26.50	70.20	60.60	71.58	58.16
Conicity index	1.32	70.20	63.60	73.14	59.34
Abdominal volume index	19.61	76.60	63.60	74.72	64.59

Weight-adjusted-waist index	0.11	70.20	66.70	74.76	60.46
Waist circumference (cm)	101.50	70.20	63.60	74.15	58.93
Neck circumference (cm)	41.75	68.10	57.60	70.49	54.83
Hip circumference (cm)	105.75	72.30	60.60	73.19	59.53
Waist-to-hip ratio	0.92	80.90	57.60	73.95	66.97
Waist-to-height ratio	0.56	83.00	60.60	75.81	70.56
Neck-to-height ratio	0.23	70.20	51.50	68.29	53.74
Body Mass Index	27.11	70.20	69.70	77.51	61.12

Discussion

In a sample of Lebanese adults, we found an alarmingly high prevalence of CMA reaching approximately 52%, with males having a notably higher prevalence (60%) than females (47.5%). Of all anthropometric indices- traditional and novel, WC and indices adjusting WC over height, i.e., AVI and WHtR revealed to be the best predictors for CMA in the total sample and among females and males, stratified. Conversely, commonly used traditional indices, including total body fat percent, neck circumference, hip circumference, and BMI, as well as novel indices adjusting WC over weight, such as C-index and WWI, had less diagnostic accuracy.

We found AVI to be a good predictor for CMA, at an optimal cut off point of 19.61 and 16.75 among males and females, respectively. Recently, Al-Ahmadi et al. (12) also identified AVI as a marker for CMA in Saudi adults and identified similar cut-offs to ours (19.1 for males and 16.3 for females). Similarly, WHtR was identified by Khaderet al. (24) as one of the best predictors for metabolic abnormalities among adult Jordanians, with a similar cut-off value of 0.5 as in our study. Recent data also support AVI and WHtR as optimal predictors for metabolic abnormalities even in non-overweight/obese adults (25). Of clinical importance, a WHtR of 0.5 is emerging as a cut-off for metabolic abnormalities among different ethnicities and even in non-overweight/obese adults (25). The value of a WHtR of 0.5 as a universal cut-off for screening for metabolic abnormalities is confirmed by the meta-analysis by Ashwellet al. (26), and large epidemiological studies in several countries (27, 28). It has been hypothesized that WHtR is a more reliable predictor than WC alone given the genetic advantage of height on cardiometabolic risk whether it be due to genetics alone or the effect of intrauterine exposures during critical period of growth and development (26). Hence, indices adjusting WC over height, i.e., AVI and WHtR, present valuable screeners for metabolic abnormalities, given their ease of measurement requiring low-cost equipment (measuring tape and stadiometer) which are widely available and that could easily be applied by all health care practitioners, in different settings including the community, primary health care, and clinical settings. Nevertheless, given the greater simplicity of its calculation. we suggest the use of WHtR in routine practice.

Furthermore, in our study sample and among stratified genders, WC was one of the best predictors of CMA, with optimal thresholds of 91.25 cm in females and 101.5 cm in males. Similar cut-off values for adult Lebanese were also reported in a national epidemiological study (91.5 cm in females and 98.5 cm in males) (29). These results suggest that optimal central

obesity cut-off points in Lebanon may be higher than those recommended by the International Diabetes Federation (IDF), i.e., 80 cm in females and 94 cm in males (1) justifying the need for nation-wide studies to elucidate the optimal cut-off to be used in screening protocols. Data from neighboring countries also suggest higher cut-off values for predicting cardiometabolic abnormalities (30, 31). All of which questions the current use of Europids criterion for WC in the diagnosis of the MetS in adult Arabs.

Clinicians have long been using BMI as a proxy measure for adiposity and screener for cardiometabolic risk (32). Our results do not support the routine use of BMI for this aim, considering the more reliable indices that we have identified. Existing meta-analyses including studies conducted among adults from various ethnicities have also questioned the clinical relevance of BMI, and support the use of WHtR in identifying adults at increased cardiometabolic risk (26).

Our finding of total body fat percent having the lowest predictive ability for CMA also challenges its clinical screening relevance. This is an interesting finding since clinicians often rely on measurement of total body fat to confer risk of cardiometabolic abnormalities. The distribution of body fat matters more than total body fat when related to cardiometabolic risk, as abdominal obesity plays a central role in the development of cardiometabolic abnormalities (33). Suggested pathways through which visceral fat negatively impacts the metabolic profile include production of inflammatory mediators and increased rate of fatty acid release (34). Hence, our results, in line with available literature, do not support the clinical use of total body fat percent in screening for cardiometabolic risk; rather, if body fat will be used, segmental analysis may be better to opt for. Nevertheless, since analyzers of visceral fat vary in quality and units of

measurement, further studies are needed to elucidate the optimal index and respective cut-off value to be used in clinical practice.

Our study was not without limitations one of which was the recruitment of the participants who responded to community announcement and thus a risk of self-selection bias could exist. Additionally, this was not a national study and although power analysis was fulfilled, future studies should be conducted to recruit a more representative sample to ensure adequate cut-offs. However, to our knowledge this is one of the few studies that have been conducted in the Middle Eastern region to identify novel ethnic specific anthropometric indices in their prediction of cardiometabolic abnormalities. Additionally, all the instruments used in this study have been validated. Furthermore, the research team recruited was highly qualified and adequately trained to collect data. Finally, total body fat percent was measured by a bioelectrical impedance analyzer which results significantly correlated with those by dual-energy X-ray absorptiometry (DXA) (35).

In conclusion, our study suggests AVI and WHtR as the most reliable predictors for CMA, along with WC, with suggested cut-off values of 19.61 and 16.75, 0.58 and 0.56, and 91.25 cm and 101.5 cm among males and females, respectively. We recommend the use of these less invasive, low-cost, easy-to-measure indices to screen widely for CMA to better manage and prevent disease and subsequent morbidity and mortality.

Acknowledgments: None

Funding: This research was funded by Zayed University under cluster R18030.

Conflicts of Interest: The authors declare no conflict of interest.

Author Contributions: Conceptualization, Myriam Abboud and Rana Rizk; Formal analysis, Rana Rizk; Funding acquisition, Myriam Abboud and Dimitrios Papandreou; Investigation, Suzan Haidar, Nadine Mahboub, and Rana Rizk; Methodology, Myriam Abboud, Suzan Haidar, Nadine Mahboub, Dimitrios Papandreou, and Rana Rizk; Project administration, Suzan Haidar and Nadine Mahboub; Supervision, Dimitrios Papandreou and Rana Rizk; Writing – original draft, Myriam Abboud; Writing – review & editing, Suzan Haidar, Nadine Mahboub, Dimitrios Papandreou, and Rana Rizk. All authors have read and agreed to the published version of the manuscript.

References

1. Alberti KG, Eckel RH, Grundy SM, Zimmet PZ, Cleeman JI, Donato KA, et al. Harmonizing the metabolic syndrome: a joint interim statement of the International Diabetes Federation Task Force on Epidemiology and Prevention; National Heart, Lung, and Blood Institute; American Heart Association; World Heart Federation; International Atherosclerosis Society; and International Association for the Study of Obesity. Circulation. 2009;120(16):1640-5.

2. Lakka HM, Laaksonen DE, Lakka TA, Niskanen LK, Kumpusalo E, Tuomilehto J, et al. The metabolic syndrome and total and cardiovascular disease mortality in middle-aged men. Journal of the American Medical Association. 2002;288(21):2709-16.

3. O'Neill S, O'Driscoll L. Metabolic syndrome: a closer look at the growing epidemic and its associated pathologies. Obesity Reviews. 2015;16(1):1-12.

4. Bahijri SM, Al Raddadi RM, Jambi H, Alaama M-NA, Ferns G. The prevalence of metabolic syndrome in an apparently healthy, normotensive and non-diabetic population in Saudi Arabia by two definitions: implications for local practice. Journal of Endocrine and Metabolic Diseases. 2013;3:18-24.

5. Hirode G, Wong RJ. Trends in the Prevalence of Metabolic Syndrome in the United States, 2011-2016. Journal of the American Medical Association. 2020;323(24):2526-8.

6. Ranasinghe P, Mathangasinghe Y, Jayawardena R, Hills AP, Misra A. Prevalence and trends of metabolic syndrome among adults in the asia-pacific region: a systematic review. BMC public health. 2017;17(1):1-9.

7. Desroches S, Lamarche B. The evolving definitions and increasing prevalence of the metabolic syndrome. Applied Physiology Nutrition and Metabolism. 2007;32(1):23-32.

8. Kolovou GD, Anagnostopoulou KK, Salpea KD, Mikhailidis DP. The prevalence of metabolic syndrome in various populations. The American journal of the medical sciences. 2007;333(6):362-71.

9. Lear SA, Gasevic D. Ethnicity and Metabolic Syndrome: Implications for Assessment, Management and Prevention. Nutrients. 2019;12(1):1-15.

10. Wang H, Liu A, Zhao T, Gong X, Pang T, Zhou Y, et al. Comparison of anthropometric indices for predicting the risk of metabolic syndrome and its components in Chinese adults: a prospective, longitudinal study. BMJ open. 2017;7(9).

11. Šoštarič A, Jenko B, Kozjek NR, Ovijač D, Šuput D, Milisav I, et al. Detection of metabolic syndrome burden in healthy young adults may enable timely introduction of disease prevention. Archives of medical science : AMS. 2019;15(5):1184-94.

12. Al-Ahmadi J, Enani S, Bahijri S, Al-Raddadi R, Jambi H, Eldakhakhny B, et al. Association Between Anthropometric Indices and Nonanthropometric Components of Metabolic Syndrome in Saudi Adults. Endocrine Society. 2022;6(6):1-12.

13. Zhang J, Zhu W, Qiu L, Huang L, Fang L. Sex- and Age-Specific Optimal Anthropometric Indices as Screening Tools for Metabolic Syndrome in Chinese Adults. International Journal of Endocrinology and Metabolism. 2018;2018:1-16.

14. National Institutes of Health. Clinical Guidelines on the Identification, Evaluation, and Treatment of Overweight and Obesity in Adults--The Evidence Report. National Institutes of Health. Obesity research. 1998;6 Suppl 2:51s-209s.

15. Mente A, Yusuf S, Islam S, McQueen MJ, Tanomsup S, Onen CL, et al. Metabolic syndrome and risk of acute myocardial infarction a case-control study of 26,903 subjects from 52 countries. Journal of the American College of Cardiology. 2010;55(21):2390-8.

16. Hsieh SD, Yoshinaga H. Do people with similar waist circumference share similar health risks irrespective of height? Tohoku Journal of Experimental Medidine. 1999;188(1):55-60.

17. Grundy SM, Cleeman JI, Daniels SR, Donato KA, Eckel RH, Franklin BA, et al. Diagnosis and management of the metabolic syndrome: an American Heart Association/National Heart, Lung, and Blood Institute Scientific Statement. Circulation. 2005;112(17):2735-52.

18. Ug A, Ceylan O. Occurrence of resistance to antibiotics, metals, and plasmids in clinical strains of Staphylococcus spp. Archives of medical research. 2003;34(2):130-6.

19. Bergman RN, Stefanovski D, Buchanan TA, Sumner AE, Reynolds JC, Sebring NG, et al. A better index of body adiposity. Obesity (Silver Spring, Md). 2011;19(5):1083-9.

20. Shidfar F, Alborzi F, Salehi M, Nojomi M. Association of waist circumference, body mass index and conicity index with cardiovascular risk factors in postmenopausal women. Cardiovascular journal of Africa. 2012;23(8):442-5.

21. Gharipour M, Sadeghi M, Dianatkhah M, Bidmeshgi S, Ahmadi A, Tahri M, et al. The cut-off values of anthropometric indices for identifying subjects at risk for metabolic syndrome in Iranian elderly men. Journal of obesity. 2014;2014:1-6.

22. Shen S, Lu Y, Qi H, Li F, Shen Z, Wu L, et al. Waist-to-height ratio is an effective indicator for comprehensive cardiovascular health. Scientific reports. 2017;7:1-7.

23. Anothaisintawee T, Sansanayudh N, Thamakaison S, Lertrattananon D, Thakkinstian A. Neck Circumference as an Anthropometric Indicator of Central Obesity in Patients with Prediabetes: A Cross-Sectional Study. BioMed research international. 2019;2019:1-8.

24. Khader YS, Batieha A, Jaddou H, Batieha Z, El-Khateeb M, Ajlouni K. Anthropometric cutoff values for detecting metabolic abnormalities in Jordanian adults. Diabetes Metabolic Syndrome and Obesity. 2010;3:395-402.

25. Wu L, Zhu W, Qiao Q, Huang L, Li Y, Chen L. Novel and traditional anthropometric indices for identifying metabolic syndrome in non-overweight/obese adults. Nutrition and Metabolism. 2021;18(1):1-10.

26. Ashwell M, Gunn P, Gibson S. Waist-to-height ratio is a better screening tool than waist circumference and BMI for adult cardiometabolic risk factors: systematic review and meta-analysis. Obesity Reviews. 2012;13(3):275-86.

27. Dang AK, Truong MT, Le HT, Nguyen KC, Le MB, Nguyen LT, et al. Anthropometric Cut-Off Values for Detecting the Presence of Metabolic Syndrome and Its Multiple Components among Adults in Vietnam: The Role of Novel Indices. Nutrients. 2022;14(19):1-14.

28. Hu J, Jiang Y, Shen H, Ding L, Xu X, Wu W. What is the best anthropometry index to evaluate the risk of metabolic abnormalities in Chinese adults? Diabetes Metabolism Research and Reviews. 2022;38(8):e3580.

29. Yamout R, Ammar W, Tabbara J, Fares S, Assi M, Sibai AM. Waist circumference cutoff points for predicting metabolic abnormalities in Lebanese adults. Archives of Medical Science-Civilization Diseases. 2019;4(1):64-71.

30. Bener A, Yousafzai MT, Darwish S, Al-Hamaq AO, Nasralla EA, Abdul-Ghani M. Obesity index that better predict metabolic syndrome: body mass index, waist circumference, waist hip ratio, or waist height ratio. Journal of obesity. 2013;2013:1-9.

31. Al-Odat AZ, Ahmad MN, Haddad FH. References of anthropometric indices of central obesity and metabolic syndrome in Jordanian men and women. Diabetes and Metabolic Syndrome: Clnical Research and Reviews. 2012;6(1):15-21.

32. Hanyuda A, Lee DH, Ogino S, Wu K, Giovannucci EL. Long-term status of predicted body fat percentage, body mass index and other anthropometric factors with risk of colorectal

carcinoma: Two large prospective cohort studies in the US. International journal of cancer. 2020;146(9):2383-93.

33. Soheilipour F, Hatami M, Salehiniya H, Alaei M. Indicators of Obesity and Cardio-Metabolic Risks: Important Consideration in Adults and Children. Current Diabetes Reviews. 2022;18(1):23-30.

34. Saad RK, Ghezzawi M, Horanieh R, Khamis AM, Saunders KH, Batsis JA, et al. Abdominal Visceral Adipose Tissue and All-Cause Mortality: A Systematic Review. Frontiers in endocrinology. 2022;13.

35. Karelis AD, Chamberland G, Aubertin-Leheudre M, Duval C, Aging EMi, Group P. Validation of a portable bioelectrical impedance analyzer for the assessment of body composition. Applied Physiology, Nutrition, and Metabolism. 2013;38(999):27-32.

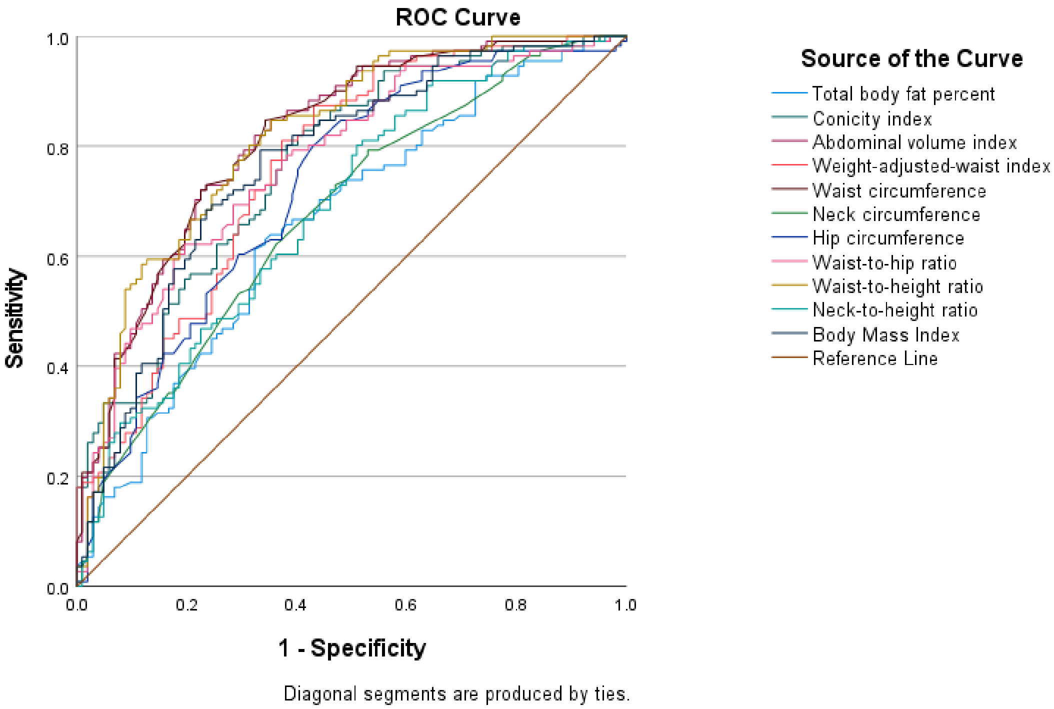

Figure 1. ROC-curves illustrating the discriminatory power of the different anthropometric indices to predict CMA in the total sample.

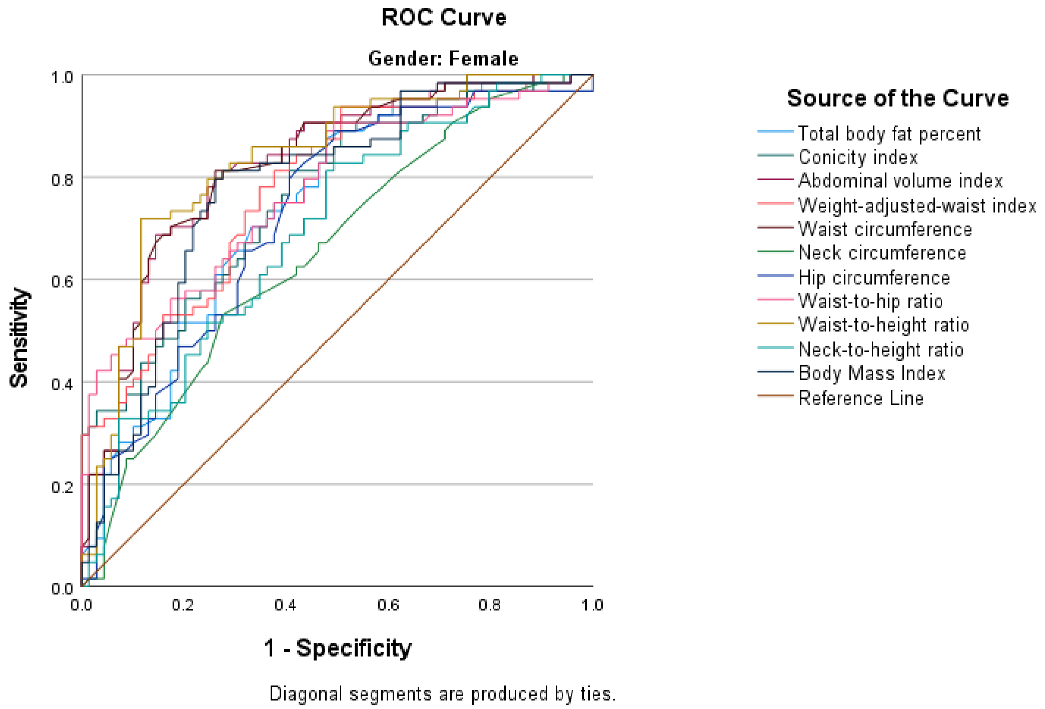

Figure 2. ROC-curves illustrating the discriminatory power of the different anthropometric indices to predict CMA among females.

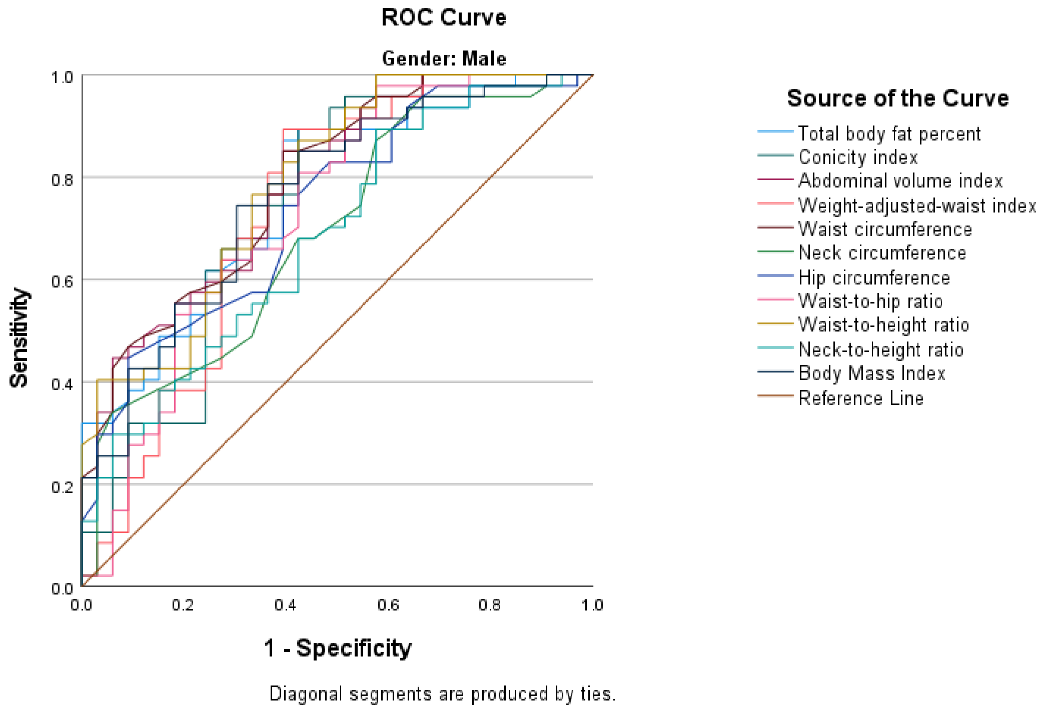

Figure 3. ROC-curves illustrating the discriminatory power of the different anthropometric indices to predict CMA among males.
