# Supplementary material for: Abdominal volume index, waist-to-height ratio, and waist circumference are optimal predictors of cardiometabolic abnormalities in a sample of Lebanese adults: A cross-sectional study
Source: PLOS Glob Public Health. 2023 Dec 21;3(12):e0002726. doi: 10.1371/journal.pgph.0002726 (PMC10734963; doi:10.1371/journal.pgph.0002726)
Supplement: Attachment — Submitted filename: Revision Letter.docx [file pgph.0002726.s007.docx]

30/07/2023

RE: PGPH-D-23-00803

Dear Editor in Chief – PLOS Global Public Health.

Thank you for your email dated, July 04 2023, enclosing the reviewers’ comments. We would like to thank the editorial board and the reviewers for the careful and thorough reading of this manuscript, and for their thoughtful comments and constructive suggestions, which we feel have substantially improved our manuscript.

We have carefully reviewed the comments and have revised the manuscript accordingly. We believe we have addressed all the major and minor comments that were raised by the reviewers and, in doing so, have crafted a paper that is more rigorous in content and clearer in presentation. Our responses are given in a point-by-point manner below.

The modifications done are shown in track changes in the manuscript. The revision has been developed in consultation with all coauthors, and each coauthor has given approval to the final form of this revision.

We hope that the responses are satisfactory and that the revised manuscript is now suitable for publication. We look forward to hearing from you soon.

Myriam Abboud

Corresponding author

*In what follows, the replies follow the reviewers’ comments in Italic. Extracts from the text are inserted between “ ”, important sentences are highlighted in* ***bold****, and* ***corrected/edited parts are underlined****.*

**Comments to the Author**

**Editor**

Journal Requirements:

1. Please send a completed 'Competing Interests' statement, including any COIs declared by your co-authors. If you have no competing interests to declare, please state "The authors have declared that no competing interests exist". Otherwise please declare all competing interests beginning with the statement "I have read the journal's policy and the authors of this manuscript have the following competing interests:"

2. Please amend your detailed Financial Disclosure statement. This is published with the article. It must therefore be completed in full sentences and contain the exact wording you wish to be published.

a. State the initials, alongside each funding source, of each author to receive each grant.
b. State what role the funders took in the study. If the funders had no role in your study, please state: “The funders had no role in study design, data collection and analysis, decision to publish, or preparation of the manuscript.”

c. If any authors received a salary from any of your funders, please state which authors and which funders.

If you did not receive any funding for this study, please simply state: “The authors received no specific funding for this work.”

3. Please provide separate figure files in .tif or .eps format only and remove any figures embedded in your manuscript file. Please also ensure all files are under our size limit of 10MB.

For more information about figure files please see our guidelines:

<https://journals.plos.org/globalpublichealth/s/figures> 
<https://journals.plos.org/globalpublichealth/s/figures#loc-file-requirement>

4. In the online submission form, you indicated that "All data are available and kept safely and confidentially with corresponding author". All PLOS journals now require all data underlying the findings described in their manuscript to be freely available to other researchers, either 1. In a public repository, 2. Within the manuscript itself, or 3. Uploaded as supplementary information.
This policy applies to all data except where public deposition would breach compliance with the protocol approved by your research ethics board. If your data cannot be made publicly available for ethical or legal reasons (e.g., public availability would compromise patient privacy), please explain your reasons by return email and your exemption request will be escalated to the editor for approval. Your exemption request will be handled independently and will not hold up the peer review process, but will need to be resolved should your manuscript be accepted for publication. One of the Editorial team will then be in touch if there are any issues.

Additional Editor Comments (if provided):

The authors need to adhere to the appropriate reporting guidelines when revising the manuscript.
Greater clarity in the reporting of the statistical analyses are required.

*Reply: In the revised version of the manuscript, we followed TRIPOD guideline, and provided more clarity in the reporting of the statistical analyses. Furthermore, and in line with Reviewer #2 comments, we added details about categorical variables, data distribution, ANCOVA, and logistic regression analyses.*

**Reviewer #1:**

The manuscript is well written though some editing is required in the grammar. The study is relevant because it established the variation and diversity in the application of markers of body adiposity and CMA in a Lebanese population. Some details is required in the methodology section. The authors should state clearly how they confirmed the nationality of the participants. Is it through any government instruments like National ID card, international passport etc. Examples of diseases affecting vitamin D metabolism should be stated and how individuals with these diseases were excluded.

*Reply: We thank the reviewer for his/her suggestions. To confirm Lebanese nationality, participants were asked to show their Lebanese identification card. Examples of diseases affecting metabolism of vitamin D were added as: chronic kidney and liver diseases. Participants were asked about previous or current medical disease diagnosis and were excluded if they reported having these diseases.*

**Reviewer #2:**

This study has an interesting and relevant research question about the relevance of various measures of adiposity to cardiometabolic abnormalities in an understudied population. It is also important that the authors have attempted to identify the optimal cut-off points for these measures, as the established ones mainly come from European populations. The statistical analysis could benefit from major changes and the aim of the study needs to be placed more clearly in the context of related research in Middle East.

Major comments:

• Introduction: I think the introduction should include more information on cardiometabolic abnormalities and their trends in the Middle East area and what gaps of research there are for such populations. Are there other similar studies in such populations?

*Reply: We thank the reviewer for his/her suggestion. Information on the cardiometabolic abnormalities and trends in the Middle East and Lebanon was added to the introduction section as follows* “***Looking at the Middle East region, the prevalence of the metabolic syndrome has increased significantly in recent years (15). This increase has been attributed to the westernization of the diet and increased intakes of fats, sugars, and salt (16). Furthermore, in contrast to trends observed in northern Europe and the USA, mortality from cardiovascular diseases has risen, where 25–45 % of total deaths in these countries are attributed to these diseases (17), with a higher prevalence among women than men (18). Lebanon, a middle-eastern country, has one of the highest estimated prevalence of metabolic syndrome among adults in the region (31.2%) (19). The growing trend towards survival in later years, in addition to the high urbanization rates, and changes in lifestyles rendered the health of the Lebanese population a major challenge***”. *Furthermore, information on the gaps of research in this population was also added as follows:* “***Studies identifying the association between anthropometric indices and cardiometabolic risks in the Middle East region are scarce. Nasreddine et al. (28) identified the optimal cutoff values of three anthropometric indices and their relation to the metabolic syndrome among Lebanese adults. The high incidence of cardiometabolic diseases in the region reinforces the need for further studies investigating the relation between several anthropometric indices and cardiometabolic risks. Furthermore, looking at ethnic and population-specific differences in the manifestation of cardiometabolic diseases in the region is warranted****”.*

*Additional references added:*

- *Chedid, R., Gannagé-Yared, M. H., Khalifé, S., Halaby, G., & Zoghbi, F. (2009). Impact of different metabolic syndrome classifications on the metabolic syndrome prevalence in a young Middle Eastern population. Metabolism, 58(6), 746-752.*
- *Amuna, P., & Zotor, F. B. (2008). Epidemiological and nutrition transition in developing countries: impact on human health and development: The epidemiological and nutrition transition in developing countries: evolving trends and their impact in public health and human development. Proceedings of the Nutrition Society, 67(1), 82-90.*
- *Yusuf, S., Reddy, S., Ôunpuu, S., & Anand, S. (2001). Global burden of cardiovascular diseases: part I: general considerations, the epidemiologic transition, risk factors, and impact of urbanization. Circulation, 104(22), 2746-2753.*
- *Fazizi, F., Esmaillzadeh, A., & Mirmiran, P. (2004). Obesity and cardiovascular disease risk factors in Tehran adults: a population-based study. EMHJ-Eastern Mediterranean Health Journal, 10 (6), 887-897, 2004.*
- *Sibai, A. M., Obeid, O., Batal, M., Adra, N., El Khoury, D., & Hwalla, N. (2008). Prevalence and correlates of metabolic syndrome in an adult Lebanese population. CVD Prevention and Control, 3(2), 83-90.*
- *Nasreddine, L., Bachir, N., Kharroubi, S., Chamieh, M. C., Mehio Sibai, A., Hwalla, N., & Naja, F. (2019). Anthropometric cutoffs for increased cardiometabolic risk among Lebanese adults: a cross-sectional study. Metabolic syndrome and related disorders, 17(10), 486-493.*

• Statistical analysis:

o Since this is a study about prediction, it would be important to follow relevant guidelines about prediction models i.e. TRIPOD (<https://pubmed.ncbi.nlm.nih.gov/25560730/> ) or STARD both for conducting the analyses and reporting them.

*Reply: The TRIPOD guideline was followed to conduct and report the analysis.*

o Lines 156-158: While discrimination is important to assess as a start, AUCs have also caveats (see for reference: <https://www.ahajournals.org/doi/10.1161/circulationaha.106.672402>), so prediction modelling should include also measures of calibration and other relevant e.g. reclassification. Please refer to the TRIPOD guidelines.

*Reply: The reviewer raises a great point in pinpointing the limitations of AUCs and suggesting the use of additional measures including the net reclassification index (NRI) statistics. NRIs for risk prediction were recommended in the TRIPOD statement (Moons et al., 2015), yet, the method was criticized in the literature (Kerr et al., 2014; Kerr, 2022; Pepe et al., 2015; Shipe et al., 2019). Specifically, Pepe et al. (2015) suggested caution in using the NRIs and recommended other measures, such as those derived from the ROC curve (c statistic) and regression coefficients. Hence, for the current analysis, we opted to retain our initial analysis and complement it- as suggested by the reviewer- with regression analyses and c statistic calculation. Furthermore, in light of the reviewer’s comment, we amended our limitations section to acknowledge the caveats of discrimination analysis (AUCs), while mentioning that it is still recommended to be used for risk prediction, as follows: “****Finally, although discrimination analysis (AUCs) has several caveats (43), it still recommended to be used for risk prediction (44, 45)****”. If the reviewer and the editor insist on complementing our analyses with NRIs, we can run these analyses and report of their results as supplements to our article.*

*Alternatively, we suggest to generate “****Expected vs Observed case ratios (E/O ratios)****” to determine model calibration, as detailed in Brooks et al. (2023).*

*Brooks JD, Mah A, Christensen RA, Arneja J, Eisen A, Chiarelli AM. Validation of the International Breast Cancer Intervention Study (IBIS) model in the High Risk Ontario Breast Screening Program: A retrospective cohort study. Genetics in Medicine. 2023 Jun 1;25(6):100820.*

*Kerr KF, Meisner A, Thiessen-Philbrook H, Coca SG, Parikh CR. Developing risk prediction models for kidney injury and assessing incremental value for novel biomarkers. Clinical journal of the American Society of Nephrology: CJASN. 2014 Aug 8;9(8):1488.*

*Kerr KF. Net Reclassification Index Statistics Do Not Help Assess New Risk Models. Radiology. 2022 Nov 15;306(3):e222343.*

*Moons KG, Altman DG, Reitsma JB, Ioannidis JP, Macaskill P, Steyerberg EW, Vickers AJ, Ransohoff DF, Collins GS. Transparent Reporting of a multivariable prediction model for Individual Prognosis or Diagnosis (TRIPOD): explanation and elaboration. Annals of internal medicine. 2015 Jan 6;162(1):W1-73.*

*Pepe MS, Fan J, Feng Z, Gerds T, Hilden J. The net reclassification index (NRI): a misleading measure of prediction improvement even with independent test data sets. Statistics in biosciences. 2015 Oct;7:282-95.*

*Shipe ME, Deppen SA, Farjah F, Grogan EL. Developing prediction models for clinical use using logistic regression: an overview. Journal of thoracic disease. 2019 Mar;11(Suppl 4):S574.*

o Line 161: Considering that you have multiple predictors, thus multiple testing, consider performing a correction for multiple testing.

*Reply: The Bonferroni correction was applied to correct for multiple testing. As we A Bonferroni correction was performed to correct the p-value over the multiple predictors used, whereby significant p-value was α / 8, i.e. < 0.00625. This clarification was added in the statistical analysis section and as a footnote in Table 2.*

o The analyses presented in Table 3 are unadjusted. It might be more informative if the p-values come from analyses adjusted for relevant factors e.g. age, socioeconomic status, and smoking. You might want to consider presenting the mean differences and their 95% CI as well.

*Reply: As recommended by the reviewer, we ran an Analysis of Covariance (ANCOVA) to display the difference in mean of the anthropometric indices between presence and absence of CMA adjusted over covariates (age, gender, education level, socioeconomic status, smoking, family history of diabetes, family history of dyslipidemia, and family history of hypertension). The adjusted means were presented in, now Table 2, in addition to the mean difference and their 95% confidence intervals.*

o When assessing prediction e.g. through discrimination (AUCs) it might be more clinically relevant to include some baseline predictors in the model e.g. age, education, SES and smoking and then assess discrimination with these predictors only and then also when adding the anthropometric markers. In this case you could also assess risk reclassification.

*Reply: We amended our statistical analysis as suggested by the reviewer. We added in Table 3 a baseline predictor model through a logistic regression analysis and calculated the c statistic, considering sociodemographic, smoking, and medical family history variables. We added also in Table 3 logistic regression analyses and c statistic calculations taking the presence/absence of CMA as the dependent variable and anthropometric indices as independent variables, adjusted over the sociodemographic and lifestyle characteristics, and family history of diseases.*

• Limitations paragraph:

o The authors mention “power analysis was fulfilled”. Could you include the power analysis in the methods section since it was done and especially since this is a relatively small study compared to other prospective studies nowadays?

*Reply: We thank the reviewer for the comment. Actually, power analysis was not conducted, in light of which the limitations section was adjusted.*

o What about loss of predictive value from potential predictors not available in the study e.g. alcohol, diet and physical activity?

*Reply: Indeed, this is a limitation of our study, which was also acknowledged in the limitations section of the revised version of the manuscript.*

• Conclusions paragraph: Here it would be appropriate to add direction for future research including large prospective cohort studies and inclusion of more predictors.

*Reply: The information required is added to the conclusion section as follows: “****To enable clinical utilization by healthcare providers, it is necessary to conduct future cohort prospective studies that encompass a broader range of variables and ensure national representativeness. These studies will yield cutoff values that can be employed effectively****”.*

Minor comments:

• Line 55: Change “low-density” to “high-density”

*Reply: This error was corrected as follows: “****Cardiometabolic abnormalities, i.e., hyperglycemia, elevated blood pressure, high triglyceride levels, low levels of high-density lipoprotein (HDL-C), and obesity (1) have long been associated with a higher risk of morbidity and mortality (2, 3)****”.*

• Line 58: Do the cited references include the potential causes (especially stress exposure) of the rise in the metabolic syndrome prevalence? If not the part of the sentence “, is rising due to alterations in the lifestyle, dietary choices, and stress exposure” can be deleted or be supported by a reference which includes this.

*Reply: The above-mentioned statement is now supported by new references, and the text now reads as follows: “****Globally, the incidence of clustered cardiometabolic abnormalities, such as the metabolic syndrome, is rising due to alterations in the lifestyle, dietary choices, and stress exposure (4-6), with global estimates being 25% and ranging from 10-84% depending on the diagnostic criteria used, age, gender, and ethnicity*** ***(7-9)****”.*

*Additional reference added:*

- *Brunner, E. J., Hemingway, H., Walker, B. R., Page, M., Clarke, P., Juneja, M., ... & Marmot, M. G. (2002). Adrenocortical, autonomic, and inflammatory causes of the metabolic syndrome: nested case-control study Circulation 106 (21): 2659-2665.*

• Line 60: References 7-8 are from approximately 15 years ago, while the sentence refers to global estimates of metabolic syndrome. Please use recent references and include the time period your estimates refer to for relevance.

*Reply: A new reference about the global estimates of metabolic syndrome.*

*Additional reference added:*

- *Saklayen, M. G. (2018). The global epidemic of the metabolic syndrome. Current hypertension reports, 20(2), 1-8.*

• Line 76: Reference 18 seems irrelevant (Occurrence of resistance to antibiotics, metals, and plasmids in clinical strains of Staphylococcus spp)

*Reply: Indeed reference 18 is irrelevant and was removed from the text and the references list.*

• Lines 154-155: It is not clear which exposures were categorical, please specify.

*Reply: The main exposure variable is the presence/absence of CMA which is categorical. The other variables are sociodemographic such as gender, marital status, education level, socioeconomic status, and employment status, medical such as presence of comorbidities, and lifestyle-related such as cigarette and/or waterpipe smoking status.*

• Line 155: t-test is a parametric method; were the assumptions met e.g. equal variances between the groups?

*Reply: The sample was normally distributed, as checked by visual inspection of the histogram of the independent variables, i.e., the anthropometric indices. Thus, parametric tests were used.*

• Since you stratified your main analyses by sex, it might be sensible to do the same for Table 1 and Table 2.

*Reply: Table 1 was stratified by gender and merged with Table 2.*

• Table 2: Since mean and median are close, it would be enough to present only mean (SD) (also min, max not necessary) and you could actually include these in Table 1.

*Reply: Means(SD) were only presented, and Table 2 was merged with Table 1.*

• Lines 178-179: “this was more common in males than females (47.5% vs. 59.8%)”, does 47.5% refer to females then? This is not clear; if this is the case please rephrase as follows: “this was more common in males than females (59.8% vs 47.5% respectively)”

*Reply: This typo was corrected.*

• Figures 1-3 could be combined in a three-panel figure.

*Reply: The figures were combined in a three-panel figure.*

6. PLOS authors have the option to publish the peer review history of their article ([what does this mean?](https://journals.plos.org/globalpublichealth/s/editorial-and-peer-review-process#loc-peer-review-history)). If published, this will include your full peer review and any attached files.

**Do you want your identity to be public for this peer review?** If you choose “no”, your identity will remain anonymous but your review may still be made public.
For information about this choice, including consent withdrawal, please see our [Privacy Policy](https://www.plos.org/privacy-policy).

Reviewer #1: No

Reviewer #2: **Yes:** Eirini Trichia

[NOTE: If reviewer comments were submitted as an attachment file, they will be attached to this email and accessible via the submission site. Please log into your account, locate the manuscript record, and check for the action link "View Attachments". If this link does not appear, there are no attachment files.]

While revising your submission, please upload your figure files to the Preflight Analysis and Conversion Engine (PACE) digital diagnostic tool, <https://pacev2.apexcovantage.com/>. PACE helps ensure that figures meet PLOS requirements. To use PACE, you must first register as a user. Registration is free. Then, login and navigate to the UPLOAD tab, where you will find detailed instructions on how to use the tool. If you encounter any issues or have any questions when using PACE, please email PLOS at [figures@plos.org](mailto:figures@plos.org). Please note that Supporting Information files do not need this step.
