# Supplementary material for: Abdominal volume index, waist-to-height ratio, and waist circumference are optimal predictors of cardiometabolic abnormalities in a sample of Lebanese adults: A cross-sectional study
Source: PLOS Glob Public Health. 2023 Dec 21;3(12):e0002726. doi: 10.1371/journal.pgph.0002726 (PMC10734963; doi:10.1371/journal.pgph.0002726)
Supplement: Attachment — Submitted filename: Response to Reviewers.docx [file pgph.0002726.s009.docx]

19/11/2023

RE: PGPH-D-23-00803R2

Dear Editor in Chief – PLOS Global Public Health.

Thank you for your email dated, November 15, 2023, enclosing the third round of comments. We deeply thank you and the reviewer for the thoughtful comments. We have reviewed all comments and have revised the manuscript accordingly. Our responses are given in a point-by-point manner below and the modifications done are shown in track changes in the manuscript. The revision has been developed in consultation with all coauthors, and each coauthor has given approval to the final form of this revision. We hope that the responses are satisfactory and that the revised manuscript is now suitable for publication.

We look forward to hearing from you soon.

Myriam Abboud

Corresponding author

*In what follows, the replies follow the reviewers’ comments in Italic. Extracts from the text are inserted between “ ”, important sentences are highlighted in* ***bold****, and* ***corrected/edited parts are underlined****.*

**Reviewers' comments:**

Reviewer #2: I would like to thank the authors once again for addressing my points in detail. In my opinion the manuscript has substantially improved, but I still have a few comments for consideration.

- Abstract-Methods: Change “c-statistic and 95% confidence intervals (CIs)” to “c-statistic [95% confidence intervals (CIs)] and calibration plots”

*Reply: We thank the reviewer for his/her comment. The line reading “c-statistic and 95% confidence intervals (CIs)” was removed and replaced by “****c-statistic [95% confidence intervals (CIs)] and calibration plots”*** in the abstract, *page 2 lines 37 and 38.*

- Abstract-Results: I do not agree with the statement “Adding anthropometric indices to sociodemographic variables improved model discrimination”. From table 3, we can see that the CIs of the c-statistic of the model with sociodemographic variables alone overlap with the CIs of the c-statistic of the model that additionally includes any of the anthropometric indices, which means that the two c-statistics do not significantly differ (which we can also see from the ROC curves). The p-values you have included in the table refer to the overall contribution of the anthropometric indices to the model rather than their incremental value over sociodemographic and lifestyle factors. I suggest instead to rewrite the statement as follows: “Adding anthropometric indices to sociodemographic variables did not significantly improve the model discrimination”.

*Reply: We thank the reviewer for his/her comment. The statement “Adding anthropometric indices to sociodemographic variables improved model discrimination”* was replaced by **“Adding anthropometric indices to sociodemographic variables did not significantly improve the model discrimination**” in the abstract, page 3 lines 47 and 48.

- Introduction – 3rd paragraph: Thank you for including more recent references. You can consider including information from this paper https://www.sciencedirect.com/science/article/pii/S0168822722007380 , which is from 2022 and highlights that “Eastern Mediterranean WHO regions had the highest prevalence across different MetS definitions”.

*Reply: We thank the reviewer for the comment. The suggested reference was used, and additional statement was added on page 4 lines 70-72:* ***“The Eastern Mediterranean region has the highest MetS prevalence across different MetS definitions that increased with the increase in the country’s level of income*.”**

**Additional Reference:**

- Noubiap, J. J., Nansseu, J. R., Lontchi-Yimagou, E., Nkeck, J. R., Nyaga, U. F., Ngouo, A. T., ... & Bigna, J. J. (2022). Geographic distribution of metabolic syndrome and its components in the general adult population: A meta-analysis of global data from 28 million individuals. *Diabetes research and clinical practice*, *188*, 109924.
- Statistical analysis: Please rewrite the sentence “In order to compare… in two groups” as “The Chi-square and Fisher exact tests were used to assess associations between categorical variables and clinical metabolic abnormalities (CMA), and the Student t-test was used to assess associations between continuous variables and CMA.”

*Reply: We thank the reviewer for his/her comment. The sentence “In order to compare between the variables and clinical metabolic abnormality (CMA), the Chi square and the Fisher exact test were used to compare between categorical values and the student t-test was used to compare continuous variables in two groups” was removed and the text now reads as follows on page 9 lines 180-182:* ***“The Chi-square and Fisher exact tests were used to assess associations between categorical variables and CMA, and the student t-test was used to assess associations between continuous variables and CMA.”***

- Thank you for providing an explanation on how you applied the FDR correction, which is helpful for the reviewers, but I think it is not necessary to be included in the manuscript. You can change the text “To correct for multiple testing…are not considered significant” to “To correct for multiple testing the Benjamini-Hochberg method was used for the analyses in the total sample and then in each sex separately (three groups of analyses with 11 tests performed in each group.” . Then in the end of the sentence you can add the appropriate reference i.e. “Benjamini Y, Hochberg Y (1995) Controlling the false discovery rate: a practical and powerful approach to multiple hypothesis testing. J R Stat Soc B 57:289–300”.

*Reply: We thank the reviewer for his/her suggestion. The text reading “To correct for multiple testing the Benjamini-Hochberg correction procedure was used as follows: the p values were ordered in an ascending way and a rank was assigned to it. The total number of tests used were 11 tests and the false discovery rate was considered 0.05. The formula used was (i/m)Q, i= the individual p-value rank, m= total number of tests, Q= the false discovery rate. The largest p value that is smaller than the critical value was considered as significant” was changed and now reads on pages 9 and 10 lines 188-190: “****To correct for multiple testing the Benjamini-Hochberg method was used for the analyses in the total sample and then in each sex separately (three groups of analyses with 11 tests performed in each group).***

- Please replace the “coefficients of the linear equation” with “calibration slope”.

*Reply: The statement “coefficients of the linear equation”* was replaced with ***“calibration slope”*** on page 10 lines 206.

- Results – sample description: Insert “were” between “and” and “employed”.

*Reply: Thank you for the correction. The word “****were****”* *was inserted between the words “and” and “employed” on page 11 line 219.*

- Replace “31.6% smoke waterpipe” with “31.7% smoke waterpipe” (as per table 1 numbers).

*Reply: The number 31.6% was replaced by* ***31.7%*** *on page 11 line 220.*

- Replace “stratifying between sex” with “stratifying by sex”.

*Reply*: *Thank you for the comment. The statement* *“stratifying between sex”* was replaced by

*“****stratifying by sex****”* *on page 13 line 230.*

- Insert “for” after “except”.

*Reply: The word “for” was inserted after the word “****except****” on page 14 line 235.*

- Results – Table 3 text: When presenting results from Table 3, please delete the sentences “When adding the anthropometric indices the discrimination improved”, “When adding the anthropometric indices, the discrimination was improved from 0.86 (sociodemographic variables) to 0.89 (WC and WHtR).” and “When adding the anthropometric indices, the discrimination was improved from 0.77 (sociodemographic variables) to 0.85 (AVI and WC)”. Instead, please add the sentence “When adding the anthropometric indices to the model with the sociodemographic variables, the discrimination did not significantly improve in the total sample or analyses stratified by sex.” right before the sentence “The discrimination power … in Figure 1”. Please see my “Abstract-Results” comment for the rationale for this change.

*Reply: Thank you for the comment. The sentences “When adding the anthropometric indices the discrimination improved” and “When adding the anthropometric indices, the discrimination was improved from 0.86 (sociodemographic variables) to 0.89 (WC and WHtR)” were replaced by the sentence “****When adding the anthropometric indices to the model with the sociodemographic variables, the discrimination did not significantly improve in the total sample or analyses stratified by sex****” on page 18 lines 258-260.*

- Table 4: This table could go to the supplementary material. It is enough to have the calibration plots in the main set of figures.

*Reply: Table 4 was removed from page 23 and added to the supplementary material (Table S2).*

- Discussion – 1st paragraph: Please replace the sentence “Finally … sociodemographic variable alone” with the sentence “Finally, adding anthropometric indices to models with sociodemographic variables did not significantly improve the discriminatory ability of the model”.

*Reply:* *We thank the reviewer for his comment. The sentence “Finally, use of anthropometric values yielded better prediction probability than sociodemographic variables alone” was replaced by “****Finally, adding anthropometric indices to models with sociodemographic variables did not significantly improve the discriminatory ability of the model****” on page 28 lines 292-294.*

- Discussion – 6th paragraph: Please add ,so the results, especially the reported prevalences and cut-off points, might not be generalizable to the general Lebanese population” after “relative small samples”. I also think that you can delete the sentence “Finally, although… for risk prediction”, because you complemented the discrimination analysis with the calibration, so you do not need to list it as a limitation.

*Reply:* *The sentence “****so the results, especially the reported prevalences and cut-off points, might not be generalizable to the general Lebanese population****”* *after “small samples” was added on page 32 lines 355 and 356. Moreover, the sentence “****Finally, although discrimination analysis (AUCs) has several caveats, it is still recommended to be used for risk prediction****” was deleted.*

- Figure 1: Panel A – Add “Overall” below “ROC curve”. Panels B and C – Remove “Gender:”. All panels- Replace “Source of the curve” with “Predictors included”. Even better, you could remove the legends from panels A and B and keep it only in Panel C and put all three plots in the same row. Apart from the sociodemographic variables it might be helpful to write the other legends as “+Body fat percent”, “+Conicity index” etc. It might be better to remove the grid from the figure background, so that the figures are cleaner.

*Reply: Figure 1 was adjusted as per the reviewer comment.*

- Figure 2 (and same for Figures S1 and S2): On the top of each plot, keep only the variable name without underscore and “observed” and include the unit in parentheses unless it’s an index or a ratio e.g. “Body fat (%)”. As suggested for Figure 1, please remove the grids to have cleaner figures.

*Reply: Figure 2 and the supplementary material were adjusted as per the reviewer comment.*
